# Interferon regulatory factor 5 suppresses epithelial‐to‐mesenchymal transition and metastasis by inducing GATA2 expression in colorectal cancer

**DOI:** 10.1002/ctm2.70077

**Published:** 2025-03-20

**Authors:** Teng Pan, Zaoqu Liu, Xue Feng, Deyu zhang, Lifeng Li, Yu Song, Qi Luo, Xiaojin Luo, Xiaohang Chen, Yao Yao, Guanglin Zhou, Jose M Vicencio, Weilong Zhang, Mingzhu Yin, Dan Wang, Jinhai Deng, Xuerui Tan, Fengxiang Wei

**Affiliations:** ^1^ Institute of Maternal and Child Medical Research Longgang District Maternity & Child Healthcare Hospital of Shenzhen City (Longgang Maternity and Child Institute of Shantou University Medical College) Shenzhen China; ^2^ Institute of Basic Medical Sciences Chinese Academy of Medical Sciences and Peking Union Medical College Beijing China; ^3^ Department of Respiratory and Critical Care Medicine Tianjin Chest Hospital Tianjin People's Republic of China; ^4^ Department of Gastroenterology Changhai Hospital Shanghai China; ^5^ Cancer center Internet Medical and System Applications of National Engineering Laboratory Zhengzhou China; ^6^ Department of Otolaryngology Head & Neck Surgery Peking University First Hospital Beijing China; ^7^ School of Basic Medical Sciences Jiamusi University Jiamusi China; ^8^ The Genetics Laboratory Longgang District Maternity and Child Healthcare Hospital of Shenzhen City Shenzhen China; ^9^ Institute of Maternal and Child Medical Research Longgang Maternity and Child Institute of Shantou University Medical College Shenzhen China; ^10^ Cancer Institute Paul O'Gorman Building University College London London UK; ^11^ Department of Hematology Lymphoma Research Center Peking University Third Hospital Beijing China; ^12^ Clinical Research Center (CRC) Medical Pathology Center (MPC) Cancer Early Detection and Treatment Center (CEDTC) Translational Medicine Research Center (TMRC) Chongqing University Three Gorges Hospital Chongqing University Wanzhou China; ^13^ Richard Dimbleby Laboratory of Cancer Research Randall Division and Division of Cancer and Pharmaceutical Sciences King's College London London UK; ^14^ Department of Cardiology First Affiliated Hospital of Shantou University Medical College Shantou China; ^15^ Human Phenome Institute Shantou University Medical College Shantou China; ^16^ Human Phenome Institute Guangdong Engineering Research Center of Human Phenome Shantou China; ^17^ Clinical Medical Research Center First Affiliated Hospital of Shantou University Medical College Shantou China

Dear Editor,

Approximately one‐third of colorectal cancer (CRC) patients develop metastasis, resulting in a mere 15% five‐year relative overall survival rate, underscoring metastasis as a pressing clinical concern. ^[^ ^1^ ^]^ Epithelial‐to‐mesenchymal transition (EMT) is a biological process that confers high plasticity to cells and is heavily implicated in cancer metastasis and progression. ^[^ ^2^, ^3^ ^]^ Interferon regulatory factor 5 (IRF5) can induce target gene expression by binding to their promoters upon translocation to the nucleus upon activation. ^[^ ^4^ ^]^ Despite its well‐known role as a key mediator of immune regulation, IRF5 has been reported to regulate the migratory capacity of cancer cells through transcription‐dependent/independent mechanisms. ^[^ ^5^ ^]^ We, here, investigated the role of IRF5 in CRC metastasis regulation.

We first performed gene set enrichment analysis on normalized IRF5 expression data from the Cancer Genome Atlas (TCGA) database. IRF5 expression was strongly associated with EMT signalling (Figure 1A). Three pairs of primary colorectal tumour tissues and liver metastatic counterparts were collected for RNA‐Seq analysis. Results showed significantly downregulated expression of IRF5 in metastatic tissues compared to primary ones, with other key regulators of metastasis showing up, including SFRP2, OLFML2B, PLVAP, IBSP, COL11A1, MMP, etc. (Figure 1B). Immunohistochemistry (IHC) analysis of tumour samples revealed significant downregulation of IRF5 in tissues with distant metastasis (Figure 1C,D). Real‐time quantitative polymerase chain reaction (RT‐qPCR) and western blot showed that IRF5 expression had a decrease in CRC tissues with metastasis compared to counterparts without metastasis, confirming decreased IRF5 expression at both transcriptional and translational levels (Figure 1E,F). Using the chi‐square test, we found an inverse correlation between IRF5 and tumour metastasis, including N stage and M stage (Table S1).

**FIGURE 1:**
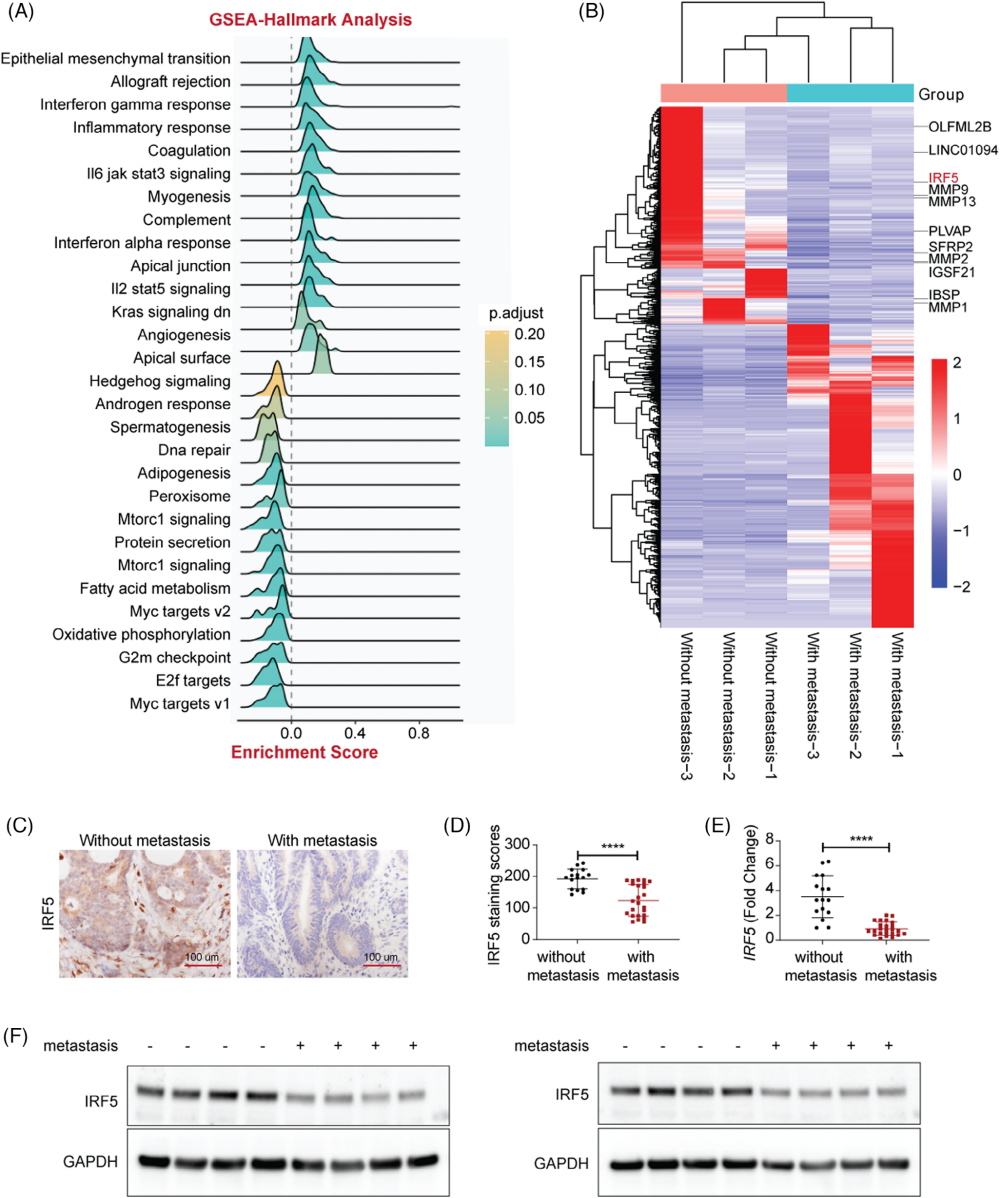
Interferon regulatory factor 5 (IRF5) is highly related to colorectal cancer metastasis. (A) The expression data for IRF5 in colorectal cancer (CRC) were obtained from the Cancer Genome Atlas (TCGA) database. Gene set enrichment analysis (GSEA) revealed that IRF5 altered the epithelial‐mesenchymal transition‐related gene expression significantly. (B) Three pairs of primary colorectal tumour tissues and liver metastatic counterparts were collected and used for RNA analysis. Heatmap illustrating differentially expressed genes. (C) Representative IRF5 immunohistochemistry (IHC) staining in CRC tissues with or without metastasis. (D) Quantification analysis of (C). (E) IRF5 mRNA levels in CRC tissues determined by real‐time quantitative polymerase chain reaction (RT‐qPCR). (F) IRF5 protein levels in CRC tissues were determined by western blotting. ****p < .0001.

To test the involvement of immune regulation, multiple public datasets were analyzed to examine the relationship between IRF5 expression and diverse immune cell types. Unexpectedly, the results showed that IRF5 was found to be significantly positively correlated with M2 macrophages (Figure S1). However, M2 macrophages have been identified to facilitate tumour metastasis. ^[^ ^6^ ^]^ Thus, the role of IRF5 involved in metastasis was not dependent on immune regulation. Further, we knocked out *IRF5* (*IKO*) in HCT116 and HCT15 cells by CRISPR‐Cas9 (Figure 2A) and analyzed transcriptional profiles of control (*NTC*) and *IKO* cells using RNA sequencing, revealing that cell migration and EMT‐related pathways were observed (Figure 2B, Figure S2 and Table S2). Functional assays also demonstrated increased invasion and migration capabilities in *IKO* groups compared to controls (Figure 2C–F). Hence, our data pointed out that IRF5‐mediated inhibition of metastasis is not tumour‐microenvironment‐dependent, yet relies on its tumour‐intrinsic role.

**FIGURE 2:**
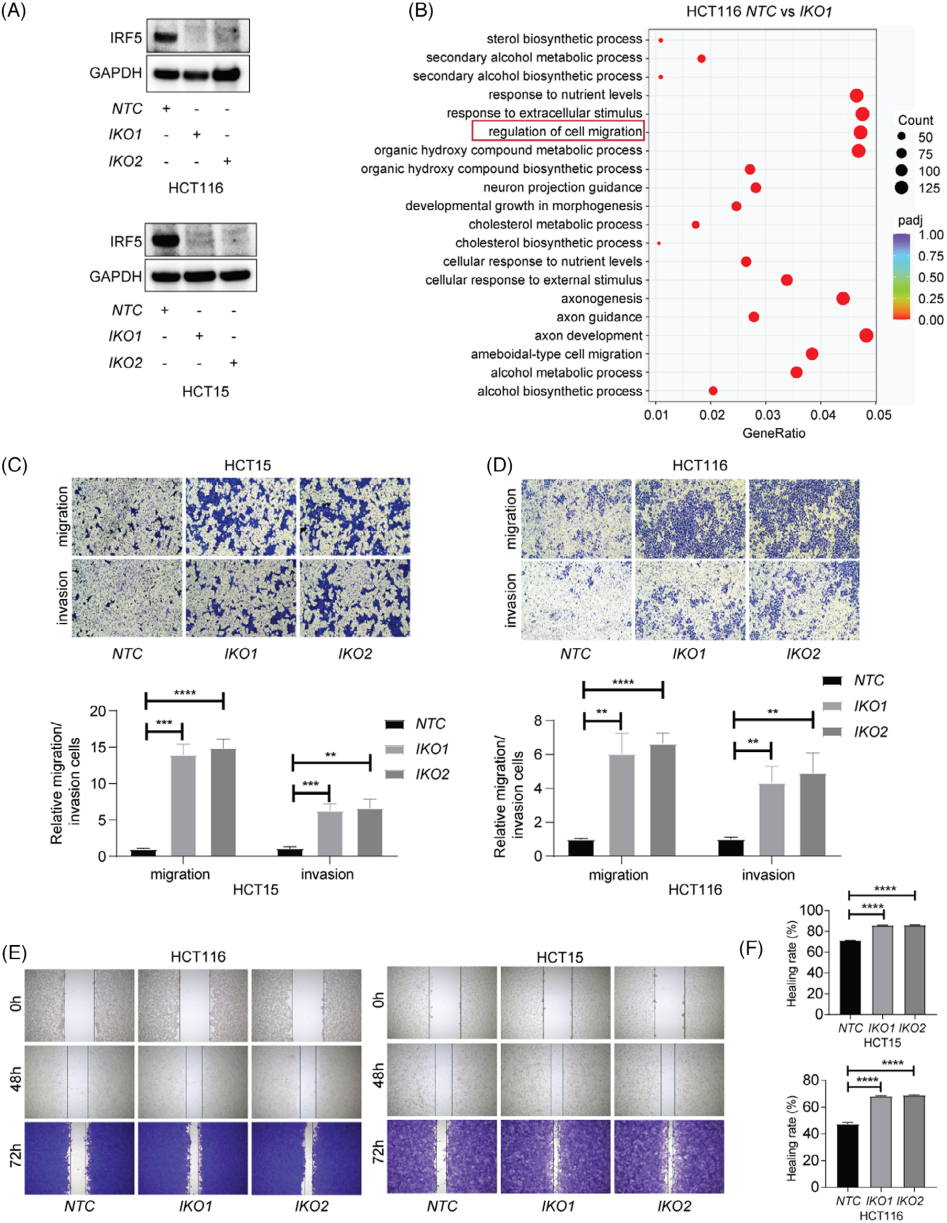
Interferon regulatory factor 5 (IRF5) inhibits colorectal cancer (CRC) cell metastasis in vitro. (A) IRF5 knockout (IKO) CRC cells were established using the CRISPR/CAS9 system. Two IRF5 knockout clones and one NTC clone were chosen. Western blotting analysis of IRF5 and GAPDH expression levels were determined by western blotting. NTC (+, ‐, ‐): NTC clone; IKO1 (‐, +, ‐): IRF5 knockout clone No.1; IKO2 (‐, ‐, +): IRF5 knockout clone No.2. (B) Total RNA from HCT116‐NTC and HCT116‐IKO1 was isolated for the RNA sequencing assay. IRF5 significantly altered the cell migration‐related gene expression in HCT116 and HCT15. (C) Transwell assays were conducted with the HCT15‐NTC and HCT15‐IKO cells to evaluate the migration and invasion capacity. (D) Transwell assays were conducted to evaluate the migration and invasion capacity with the HCT116‐NTC and HCT116‐IKO cells. (E) Wound‐healing assays were performed with HCT15‐NTC, HCT15‐IKO, HCT116‐NTC and HCT116‐IKO cells. (F) Quantification analysis of (E). **p < .01; ***p < .001; ****p < .0001. NTC: Nontargeting control. IKO: IRF5 knockout.

We next identified 84 differentially expressed genes (DEGs) associated with cell migration in HCT15 and 134 in HCT116, with 34 overlapping genes. Notably, GATA2 was identified as significantly upregulated (Figure 3A and Tables S3 and S4). Validation of GATA2 mRNA and protein levels showed substantial upregulation in *IKO* groups (Figure 3B‐C and S3A,B). IHC staining, RT‐qPCR and western blot analysis of tumour samples also indicated an increased GATA2 in metastatic tissues (Figure 3D,E and S3C,D). Moreover, IRF5 levels in patient tissues were negatively correlated with GATA2 levels (Figure 3F). Clinically, high GATA2 expression was positively correlated with advanced T stage, N stage, and M stage (Table S5).

**FIGURE 3:**
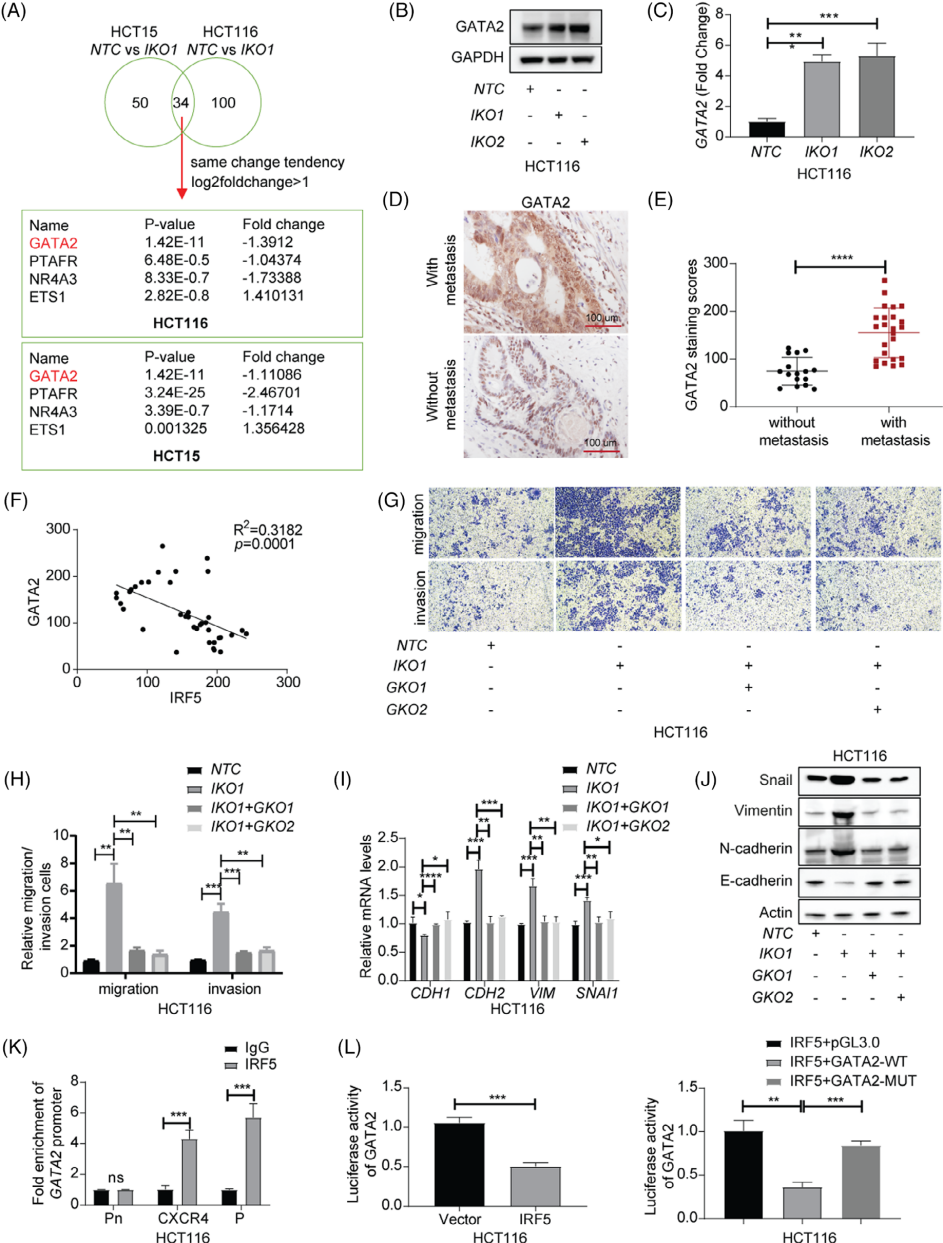
GATA2 is required for suppressing interferon regulatory factor 5 (IRF5)‐mediated colorectal cancer (CRC) invasion, migration, and epithelial‐to‐mesenchymal transition (EMT) process in vitro. (A) Analysis of overlapped cell migration‐related genes after IRF5 knockout between HCT116 and HCT15. (B) GATA2 and GAPDH expression in IKO and NTC CRC cells was verified by western blotting. (C) GATA2 mRNA levels in IKO and NTC CRC cells were determined by real‐time quantitative polymerase chain reaction (RT‐qPCR). (D) Representative GATA2 immunohistochemistry (IHC) staining in CRC tissues with or without metastasis. (E) Quantification analysis of (D). (F) Correlations of IRF5 and GATA2 protein (cohort of 40 CRC patients). (G) Transwell assays were performed in NTC, IKO and DKO CRC cells. (H) Quantification analysis of (G). (I) RT‐qPCR were performed in NTC, IKO1 and DKO CRC cells. (J) EMT‐related markers were verified by western blotting. (K) ChIP was conducted to determine the binding between IRF5 and the GATA2 promoter in HCT116 cells. RT‐qPCR experiment was conducted using primers against GATA2 promoter regions indicated. Pn is the negative control and is in the region where is out of the promoter of GATA2. CXCR4 is a positive control as IRF5 was reported to transcriptionally regulate CXCR4. P is the targeted site. (L) Analysis of the luciferase activation of the wild‐type (WT) GATA2 promoter‐driven luciferase reporter in vector and IRF5‐overexpressing CRC cells (left panel) or WT and mutant (MUT) GATA2 promoter‐driven luciferase reporters in IRF5‐overexpressing CRC cells (right panel).

To investigate whether IRF5 inhibits metastasis and EMT process in CRC via repressing GATA2 induction, we established double knock‐out cell models (*DKO*) by knocking out *GATA2* (*GKO*) (Figure S3E). Deletion of GATA2 in IRF5‐deficient cells significantly reduced their invasive and migratory capabilities (Figure 3G,H and Figure S3F–I). RT‐qPCR and Western Blot analysis demonstrated a reversal in the expression of EMT‐related markers upon GATA2 knockout (Figure 3I,J and S4A,B). Next, we wondered whether IRF5‐mediated GATA2 suppression was transcriptionally‐dependent. JASPAR database predictions showed that there might be a binding between IRF5 and ‐791 to ‐778 bp region of the GATA2 promoter (Figure S4C). Further, chromatin immunoprecipitation (ChIP) assays confirmed significant enrichment of the GATA2 promoter by anti‐IRF5 antibody (Figure 3K and Figure S4D). Furthermore, a dual luciferase reporter assay demonstrated that IRF5 overexpression significantly decreased wild‐type GATA2 promoter activity (Figure 3L and Figure S4E).

For further evaluation of IRF5's role in metastasis of CRC in vivo, we transplanted luciferase‐labeled HCT15 *NTC*, *IKO*, and *DKO* cells into the spleens of nude mice. The *IKO* group showed increased luciferase signal intensities and reduced overall survival, both of which were mitigated by *GATA2* knockout (Figure 4A–C). Additionally, the *IKO* group developed more metastatic nodules compared to the *NTC* or *DKO* groups (Figure 4D).

**FIGURE 4:**
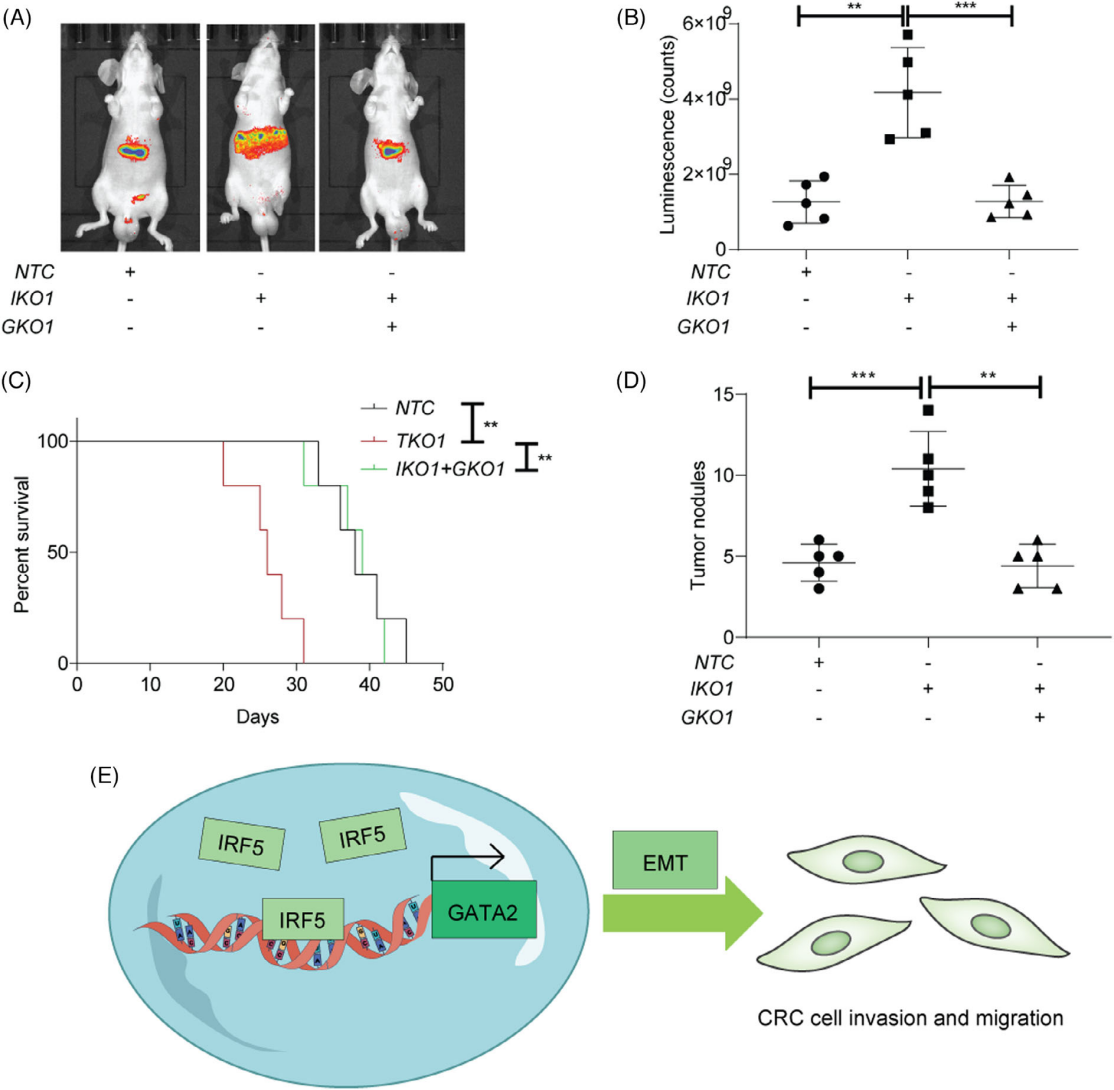
Interferon regulatory factor 5 (IRF5) suppresses the tumour metastasis of colorectal cancer (CRC) via altering GATA2 expression in vivo. (A) Nude mice were intrasplenically transplanted with luciferase‐labelled HCT15 NTC, IKO and DKO cells, with regular bioluminescence imaging to monitor tumour growth. (B) Statistical analysis of luminescence among groups. (C) Kaplan‐Meier survival curves of nude mice were analyzed. (D) Ultrasound scan was performed on 10 random regions of each mouse liver, and metastasis tumour numbers were calculated. (E) Schematic diagram shows that IRF5 decreases CRC cell migration by inhibiting epithelial‐to‐mesenchymal transition (EMT) by suppressing the transcriptional activity of GATA2. **p < .01; ***p < .001. NTC: Non‐targeting control. IKO: IRF5 knockout. GKO: GATA2 knockout.

Metastatic CRC remains a formidable challenge with a dismal prognosis.^[^ ^7^ ^]^ Therefore, it is urgent for the research community to find new targets to be specifically against metastasis for clinical application.^[^ ^8^ ^]^ Genetic ablation of *IRF5* showed that IRF5 affected the migration and invasion phenotype in CRC, highlighting that IRF5 contributed to tumour‐intrinsic functions. Further RNA sequencing data uncovered GATA2 as the downstream target of IRF5. PCR and ChIP analyses proved that IRF5 transcriptionally regulated GATA2 expression. Notably, GATA2 is a potent driver of metastasis.^[^ ^9^, ^10^ ^]^ By exploiting *DKO* cell models, our data further supported that GATA2 could mediate IRF5‐induced suppression of EMT. Collectively, we found a novel mechanism by which IRF5 exerts tumour‐intrinsic roles in regulating EMT machinery to inhibit metastasis (Figure 4E). However, further studies should be performed focusing on how GATA2 regulates EMT‐associated targets. Given the multiple expression patterns and pleiotropic roles of IRF5, designing antagonists or agonists based on IRF5‐GATA2 signalling will be the next step in the investigation for better clinical application.

## AUTHOR CONTRIBUTIONS

Conception and design: Jinhai Deng, Xuerui Tan, Fengxiang Wei and Teng Pan. Writing the manuscript: Teng Pan and Jinhai Deng. Data analysis and performance of most of the experiments: Teng Pan. Data collection: Zaoqu Liu, Lifeng Li, Deyu Zhang, Qi Luo, Xiaojin Luo, Xiaohang Chen, Yao Yao, Guanglin Zhou, Jose M Vicencio, Weilong Zhang, Mingzhu Yin, Dang Wang and Richard Beatson.

## CONFLICT OF INTEREST STATEMENT

The authors declared no conflict of interest.

## ETHICS STATEMENT

The study was authorized by the Institutional Ethics Committees of Chongqing University Three Gorges Hospital.

## Supporting information



Supporting Information

## Data Availability

Datasets are available from the corresponding author.
