# Supplementary material for: Interferon regulatory factor 5 suppresses epithelial‐to‐mesenchymal transition and metastasis by inducing GATA2 expression in colorectal cancer
Source: Clin Transl Med. 2025 Mar 20;15(3):e70077. doi: 10.1002/ctm2.70077 (PMC11925600; doi:10.1002/ctm2.70077)
Supplement: Supplementary file 1 — Supporting Information [file CTM2-15-e70077-s001.docx]

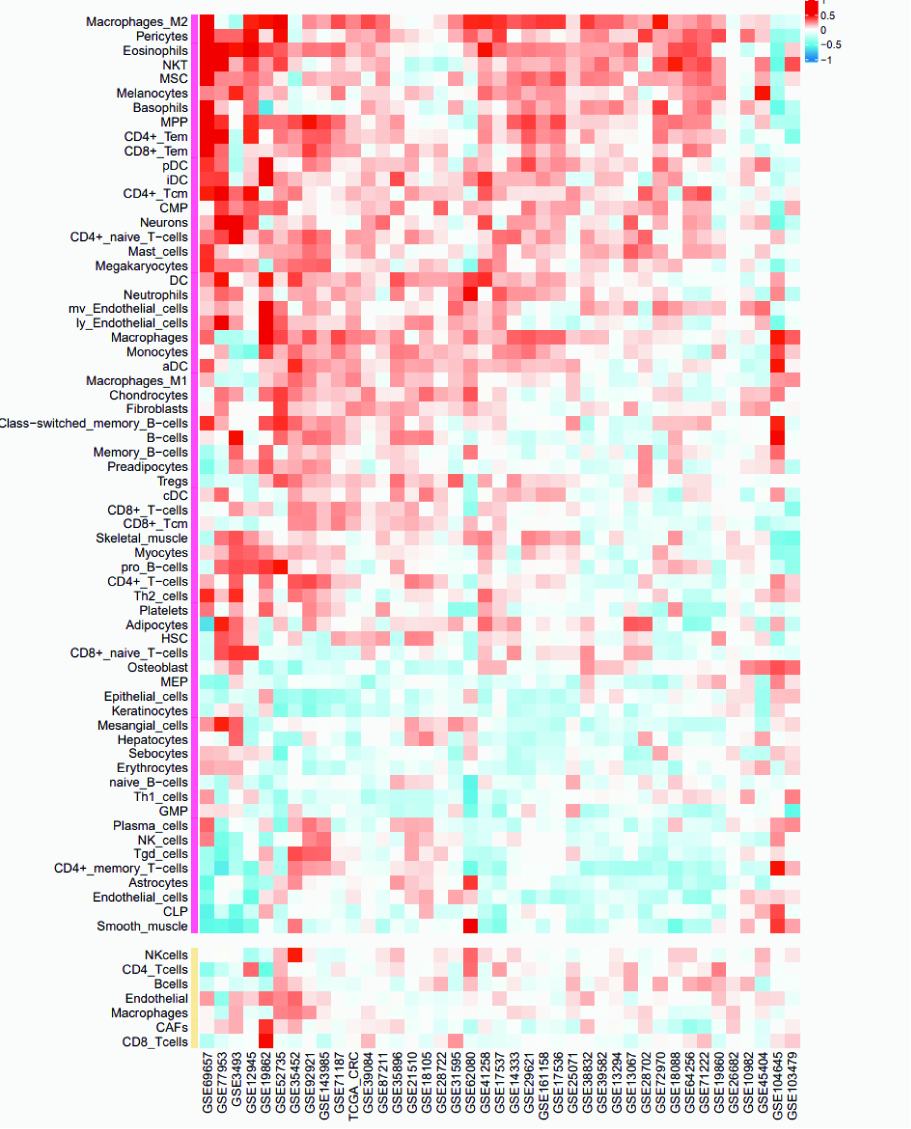


**Fig.S1 IRF5 expression was highly positively correlated with M2 infiltration.** Distribution of immune cell infiltration among multiple public datasets obtained by hierarchical clustering. The analysis was conducted to analyze the correlations between IRF5 and immune infiltration across varies CRC datasets via BEST tool (https://rookieutopia.com/). The horizontal axis listed multiple databases; the vertical axis listed the different immune cell types. Each small square represented the correlation coefficient between IRF5 expression and levels of immune cell infiltration. The color represents the Pearson correlation coefficient. High dense of red indicates high positive co-efficiency, high dense of blue indicates high negative co-efficiency.


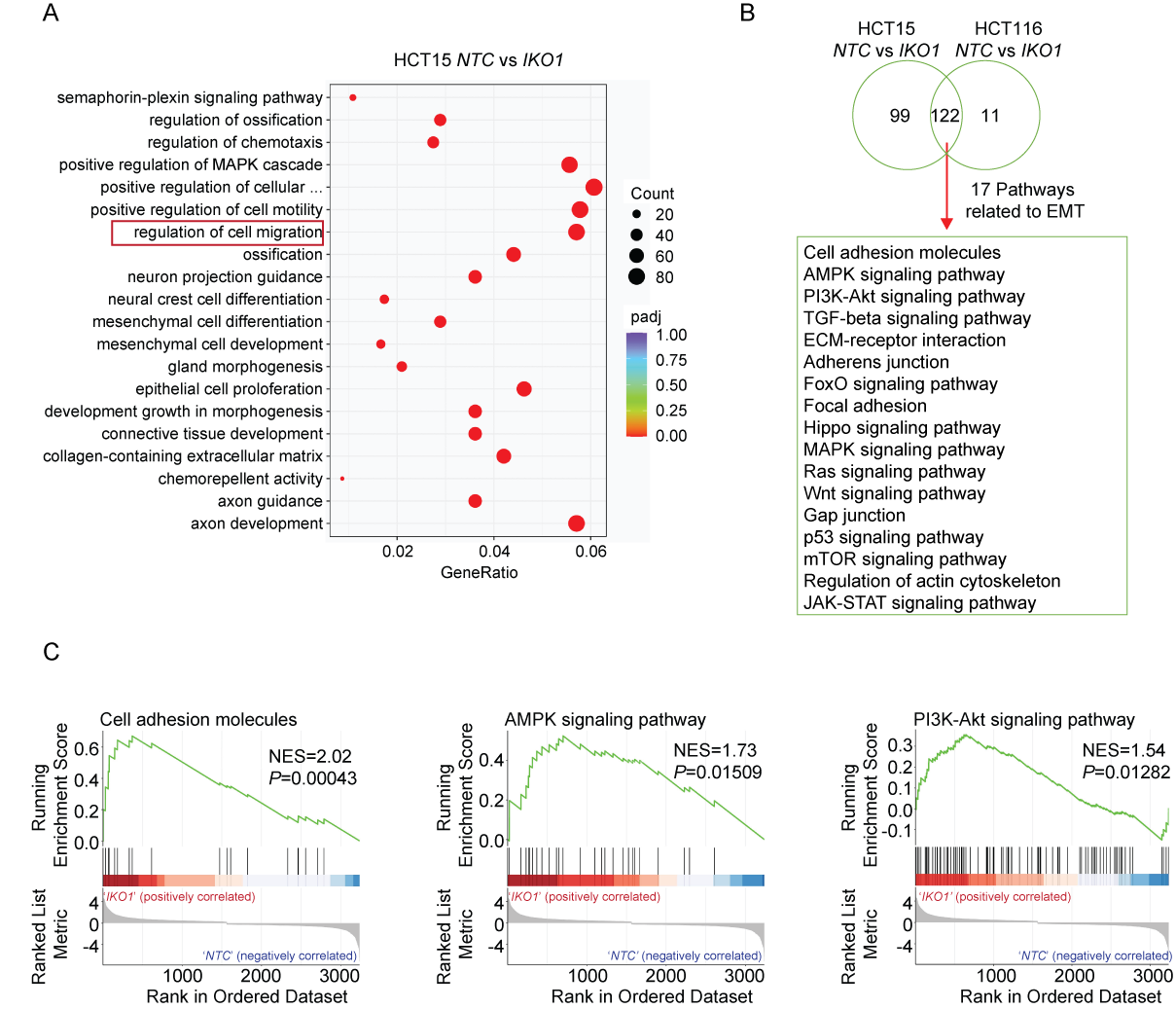


**Fig.S2 IRF5 inhibits CRC cells metastasis.** (A) Total RNA from HCT15-*NTC* and HCT15-*IKO1* was isolated for the RNA sequencing assay. IRF5 is significantly altered the cell migration related gene expression in HCT116 and HCT15. (B) There are 122 pathways commonly shared between HCT15 and HCT116. Among them, 17 pathways related to EMT were observed. (C) Enrichment of Cell adhesion molecules, AMPK signaling pathway and PI3K-Akt signaling pathway by Gene set enrichment analysis (GSEA) analysis. Abbreviation: *NTC*: Non-targeting control. *IKO*: IRF5 knockout.


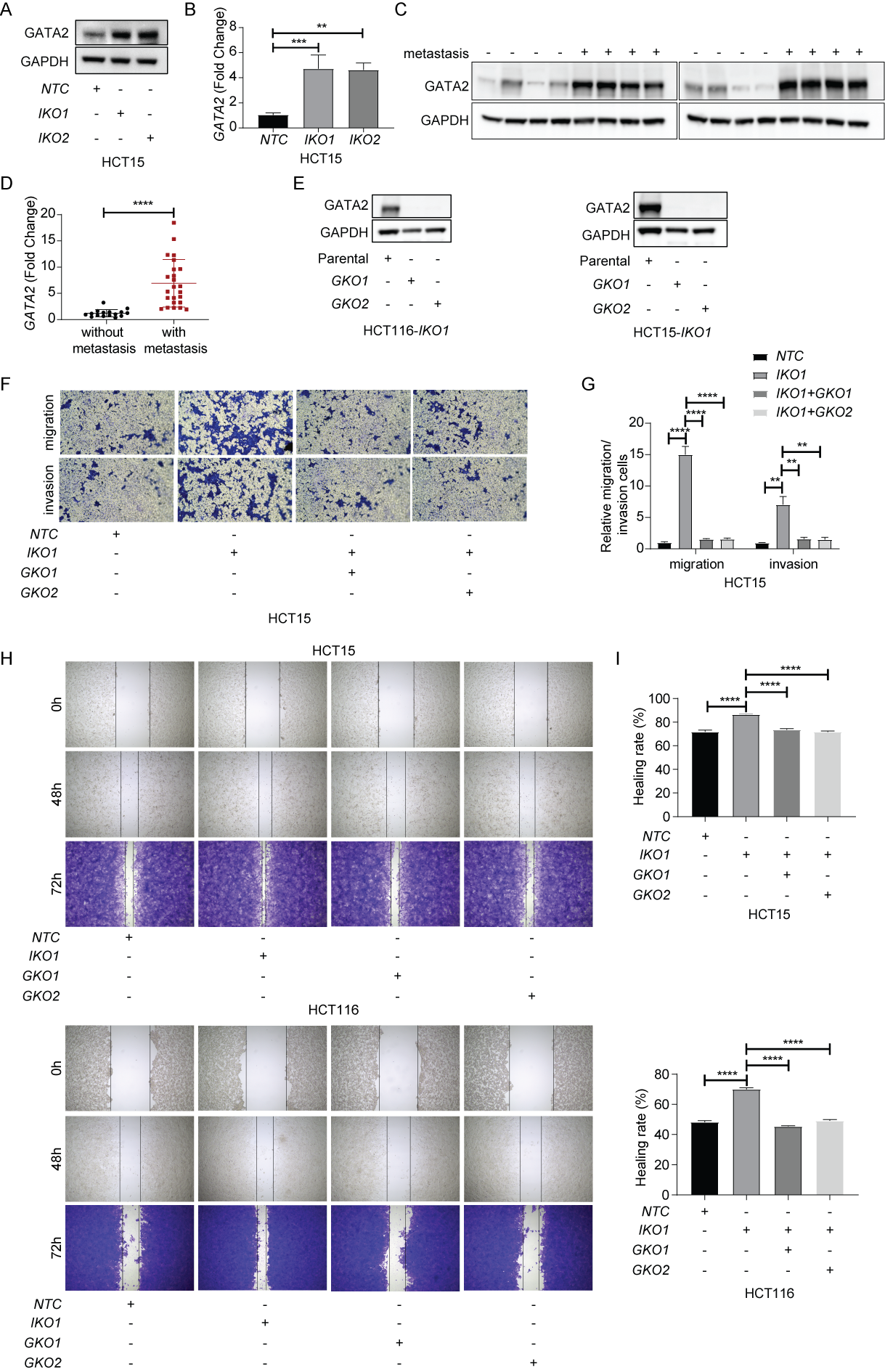


**Fig.S3 GATA2 is required for suppressing IRF5-mediated CRC migration.** (A) GATA2 and GAPDH expression in *IKO* and *NTC* CRC cells was verified by western blotting. (B) GATA2 mRNA levels in *IKO* and *NTC* CRC cells were determined by RT-qPCR. (C) Western blotting analysis of GATA2 expression in CRC tissues. (D) The expression of GATA2 mRNA levels in CRC tissues. (E) *IRF5* and *GDF15* double knockout (*DKO*) CRC cells were established using CRISPR/CAS9 system. GATA2 and GAPDH expression in indicated cell lines were determined by western blotting. (F) Transwell assays were performed in *NTC*, *IKO* and *DKO* CRC cells. (G) Quantification analysis of (F). (H) Wound-healing assays were conducted in indicated HCT15 and HCT116 cells. (I) Quantification analysis of (H). **p < 0.01; ***p < 0.001; ****p < 0.0001. Abbreviation: *NTC*: Non-targeting control. *IKO*: IRF5 knockout. *GKO*: GATA2 knockout. *DKO*: *IRF5* and *GATA2* double knockout.


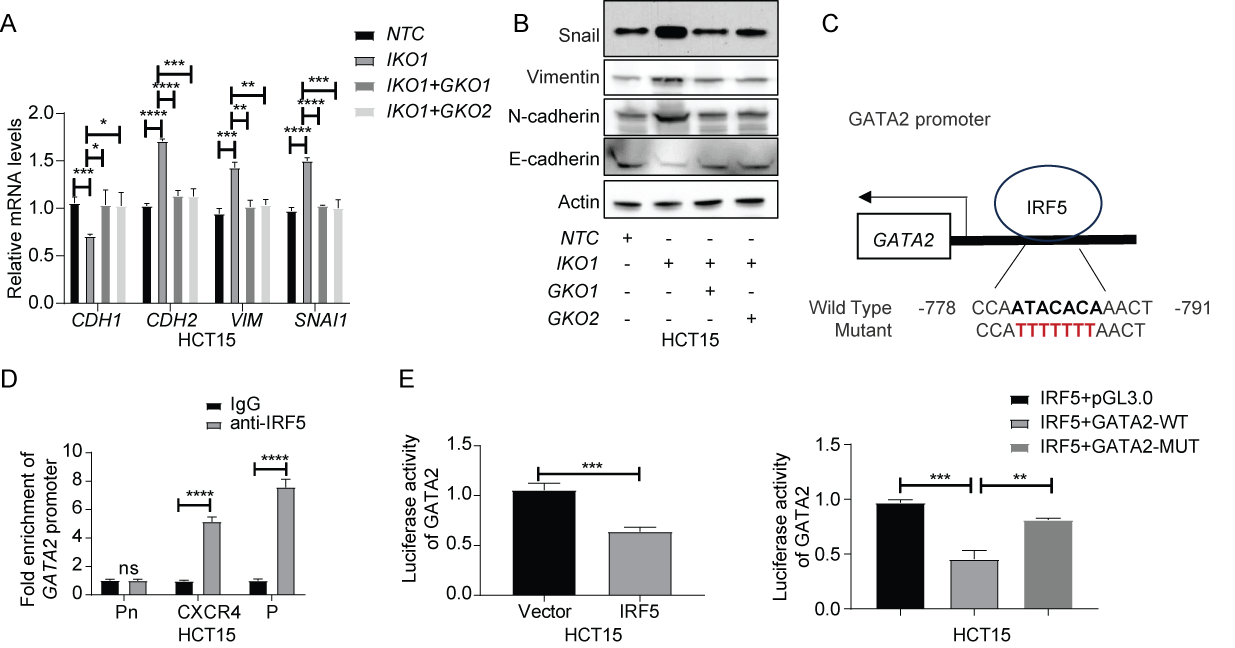


**Fig.S4 GATA2 is required for suppressing IRF5-mediated EMT process.** (A) RT-qPCR were performed in *NTC*, *IKO* and *DKO* CRC cells. (B) EMT-related markers were verified by western blotting. (C) Diagram of the GATA2 promoter, with the IRF5 binding motif in the GATA2 promoter. (D) ChIP was conducted to determine the binding between IRF5 and the GATA2 promoter in HCT15 cells. RT-PCR experiment was conducted using primers against GATA2 promoter regions indicated. (E) Analysis of the luciferase activation of the wild-type (WT) GATA2 promoter-driven luciferase reporter in vector and IRF5-overexpressing CRC cells (left panel) or WT and mutant (MUT) GATA2 promoter-driven luciferase reporters in IRF5-overexpressing CRC cells (right panel). *p < 0.05; **p < 0.01; ***p < 0.001; ****p < 0.0001. Abbreviation: *NTC*: Non-targeting control. *IKO*: IRF5 knockout. *GKO*: GATA2 knockout. *DKO*: *IRF5* and *GATA2* double knockout.

Table S1：Association between IRF5 expression and clinicopathological features of patients with colorectal cancer

|  |  | IRF5 expression | |  | |  |
| --- | --- | --- | --- | --- | --- | --- |
|  | Total | Low | High |  | |  |
|  | (n = 40) | (n = 20) | (n = 20) | *P* value | |  |
| Gender |  |  |  | 0.7515 | |  |
| Male | 21 | 10 | 11 |  | |  |
| Female | 19 | 10 | 9 |  | |  |
| Age(year) |  |  |  | 0.5250 | |  |
| <60 | 18 | 10 | 8 |  | |  |
| ≥60 | 22 | 10 | 12 |  | |  |
| T stage |  |  |  | 0.3404 | |  |
| T1-T2 | 18 | 7 | 11 |  | |  |
| T3-T4 | 22 | 13 | 9 |  | |  |
| N stage |  |  |  | 0.0252* | |  |
| N0 | 17 | 5 | 12 |  | |  |
| N1+N2+N3 | 22 | 15 | 8 |  | |  |
| M stage |  |  |  | 0.0090* | |  |
| M0 | 25 | 8 | 17 |  | |  |
| M1 | 15 | 12 | 3 |  | |  |
|  |  |  |  |  |  |  |
| ^*^*P*<0.05 | | |  |  |  | |

Table S2: 122 pathways commonly shared between HCT15 and HCT116

| Cell adhesion molecules |
| --- |
| Transcriptional misregulation in cancer |
| Toxoplasmosis |
| Efferocytosis |
| Dilated cardiomyopathy |
| Neuroactive ligand-receptor interaction |
| AMPK signaling pathway |
| Human T-cell leukemia virus 1 infection |
| Cytoskeleton in muscle cells |
| PI3K-Akt signaling pathway |
| Parathyroid hormone synthesis, secretion and action |
| cAMP signaling pathway |
| Axon guidance |
| Proteoglycans in cancer |
| TGF-beta signaling pathway |
| Hypertrophic cardiomyopathy |
| ECM-receptor interaction |
| Colorectal cancer |
| Estrogen signaling pathway |
| C-type lectin receptor signaling pathway |
| Adherens junction |
| Apoptosis |
| FoxO signaling pathway |
| Cushing syndrome |
| Human papillomavirus infection |
| Pathways in cancer |
| Chronic myeloid leukemia |
| Cholinergic synapse |
| Osteoclast differentiation |
| Relaxin signaling pathway |
| Motor proteins |
| Focal adhesion |
| Aldosterone synthesis and secretion |
| Protein digestion and absorption |
| Platelet activation |
| Cytokine-cytokine receptor interaction |
| Chemical carcinogenesis - receptor activation |
| Huntington disease |
| Hippo signaling pathway |
| MAPK signaling pathway |
| Insulin signaling pathway |
| Glucagon signaling pathway |
| Insulin resistance |
| Small cell lung cancer |
| Hepatocellular carcinoma |
| Autophagy - animal |
| Taste transduction |
| Prion disease |
| Hepatitis B |
| Ras signaling pathway |
| Gastric cancer |
| Arrhythmogenic right ventricular cardiomyopathy |
| Ovarian steroidogenesis |
| Oxytocin signaling pathway |
| Sphingolipid signaling pathway |
| Inflammatory mediator regulation of TRP channels |
| Adrenergic signaling in cardiomyocytes |
| cGMP-PKG signaling pathway |
| Wnt signaling pathway |
| Chemokine signaling pathway |
| Hematopoietic cell lineage |
| Cellular senescence |
| Gap junction |
| Parkinson disease |
| p53 signaling pathway |
| Apelin signaling pathway |
| Tight junction |
| Signaling pathways regulating pluripotency of stem cells |
| Thyroid hormone signaling pathway |
| Fluid shear stress and atherosclerosis |
| Glutamatergic synapse |
| MicroRNAs in cancer |
| Morphine addiction |
| Viral carcinogenesis |
| Melanoma |
| Tuberculosis |
| Neurotrophin signaling pathway |
| Growth hormone synthesis, secretion and action |
| Diabetic cardiomyopathy |
| mTOR signaling pathway |
| Serotonergic synapse |
| Mineral absorption |
| Pathogenic Escherichia coli infection |
| Herpes simplex virus 1 infection |
| Renin secretion |
| Lipid and atherosclerosis |
| Epstein-Barr virus infection |
| Purine metabolism |
| Melanogenesis |
| Regulation of actin cytoskeleton |
| Carbon metabolism |
| Breast cancer |
| Phospholipase D signaling pathway |
| Alcoholism |
| Metabolic pathways |
| GnRH signaling pathway |
| Hepatitis C |
| Human cytomegalovirus infection |
| Synaptic vesicle cycle |
| Dopaminergic synapse |
| Vascular smooth muscle contraction |
| Pathways of neurodegeneration - multiple diseases |
| Rap1 signaling pathway |
| Calcium signaling pathway |
| Biosynthesis of cofactors |
| Amyotrophic lateral sclerosis |
| Phosphatidylinositol signaling system |
| Inositol phosphate metabolism |
| Kaposi sarcoma-associated herpesvirus infection |
| Endocytosis |
| Measles |
| ErbB signaling pathway |
| Th17 cell differentiation |
| Basal cell carcinoma |
| Nucleotide metabolism |
| Fc gamma R-mediated phagocytosis |
| Alzheimer disease |
| Shigellosis |
| JAK-STAT signaling pathway |
| Th1 and Th2 cell differentiation |
| Phagosome |
| B cell receptor signaling pathway |

Table S3：The same *Regulation of cell migration*-related genes after IRF5 knockout between HCT116 and HCT15.

| Cell type | Gene set |
| --- | --- |
| HCT15 | MDK, IGFBP5, PTAFR, CD74, PRKD1, GPER1, AKT3, GATA2, PPP3CA, VIL1, JUN, MAP2, NEDD9, CSF1, RHOB, SEMA3A, LGR6, NXA1, SEMA4C, SEMA3G, FGF9, SEMA3F, BMP4, MYO1C, RAC2, IL6R, NR4A3, NOTCH1, NRP2, SEMA4A, PTN, ZNF703, MET, HDAC7, GATA3, BCL2, IL1R1, SEMA4F, ATOH8, GLIPR2, TRPM4, CAV1, STK39, THBS1, F2RL1, NRP1, FGF18, TTLL6, SEMA3B, LRP1, EDN1, EPB41L5, SEMA5A, CPNE3, SMOC2, PDGFRB, LPAR1, PODXL, SCARB1, IRS2, PLK2, BMPR2, HYAL1, SEMA6B, SEMA6C, SOX9, PAK1, RNF207, LGALS3, PDGFC, ETS1, INSR, TDGF1, TRIM32, JAK2, DOCK7, TGA2B, CAMK1D, PTP4A1, F2R, NTN1, PDGFB, ITGA3, VEGFB |
| HCT116 | FGFBP1, TGFB2, SEMA3B, SEMA3C, GATA2, SEMA3A, PTP4A1, SEMA3D, TIAM1, SMURF2, NRP1, ATP8A1, F2RL1, FGF2, FAM83H, BMPR2, SMAD3, SYNE2, PLA2G7, SOX9, HYAL1, ETS1, LGMN, SEMA5B, CEMIP, ANXA1, TERT, ADGRA2, TGFB1, AKT3, NR4A3, GPER1, ADAM10, RET, ROCK2, MAP3K3, PLK2, ITGA2, ITGA3, SPHK1, CORO1A, MDM2, DAPK2, XBP1, PTAFR, BCAS3, IGF1R, SEMA6C, NRP2, HSPB1, CSF1, ONECUT2, SCARB1, CXCL8, CTSH, EPHA1, FGF18, RREB1, SDCBP, SH3RF2, GRN, HMOX1, TNFSF18, DYSF, WNT11, DOCK4, PLCG1, PDGFC, ZNF268, SEMA4C, TRIP6, CARMIL1, STK39, BMP4, SEMA3E, LGR6, SPAG9, HDAC7, LAMB1,  S100A14, NSMF, ANGPT4, BCAR1, MYLK, SEMA6B, MGAT5, STAT3, FGFR1, ITGAV, SASH1, STX4, RIPOR1, GCNT2, HRAS, CCBE1, HIF1A, NUMB, ITGA2B, TMSB4X, WNK1, ENPP2, PLPP3, MAPK14, ITGA5, SPARC, IGFBP5, CARMIL2, ADAMTS1, FGF9, MYO1C, FLNA, EGFR, KITLG, MMP9, GPSM3, DMTN, VSIR, RUFY3, MYADM, AGER, RDX, TGFBR2, LRRC15, SEMA4D, ZC3H12A, MIR210, HDAC4, ZNF580, CD151, ATM, ZP3, GLIPR2, PTGS2, SSH1 |
| Overlapped genes between HCT116 and HCT15 | IGFBP5, PTAFR, GPER1, AKT3, GATA2, CSF1, SEMA3A, LGR6, ANXA1, SEMA4C, FGF9, BMP4, MYO1C, NR4A3, NRP2, HDAC7, GLIPR2, STK39, F2RL1, NRP1, FGF18, SEMA3B, SCARB1, PLK2, BMPR2, HYAL1, SEMA6B, SEMA6C, SOX9, PDGFC, ETS1, ITGA2B, PTP4A1, ITGA3 |

Table S4：Overlapped cell migration-related genes after IRF5 knock out between HCT116 and HCT15.

| HCT15 | | | | | | | |  |
| --- | --- | --- | --- | --- | --- | --- | --- | --- |
| gene_name | NTC_1 | NTC_2 | NTC_3 | IKO_1 | IKO_2 | IKO_3 | log2FoldChange |  |
| **PTAFR** | **32.42108** | **62.07266** | **48.88488** | **254.6409** | **264.3769** | **279.7641** | **-2.467010078** |  |
| **NR4A3** | **70.07137** | **86.55202** | **84.60845** | **146.6415** | **187.4064** | **210.5973** | **-1.171404595** |  |
| **GATA2** | **1020.741** | **965.1862** | **849.8449** | **1932.1** | **1850.638** | **2340.314** | **-1.110858105** |  |
| **ETS1** | **42.87949** | **71.68955** | **43.24432** | **23.7797** | **26.77235** | **11.35574** | **1.356427609** |  |
| IGFBP5 | 9.412572 | 21.85657 | 15.0415 | 181.3202 | 161.7496 | 236.4058 | -3.62564 |  |
| CSF1 | 642.1466 | 677.5537 | 598.8398 | 277.4298 | 369.2353 | 301.4432 | 1.01861 |  |
| GPER1 | 283.423 | 238.6737 | 255.7055 | 636.1069 | 641.4208 | 703.0234 | -1.35017 |  |
| AKT3 | 93.07988 | 94.42038 | 123.1523 | 325.98 | 327.9612 | 308.6696 | -1.63086 |  |
| SEMA3A | 1083.492 | 1108.565 | 735.1534 | 496.4012 | 263.2614 | 325.187 | 1.430627 |  |
| LGR6 | 377.5487 | 255.2847 | 321.5121 | 681.6846 | 611.3019 | 729.8642 | -1.08639 |  |
| F2RL1 | 3438.726 | 4093.299 | 3649.445 | 2544.427 | 2886.951 | 2673.76 | 0.464529 |  |
| STK39 | 846.0856 | 1299.155 | 1142.214 | 663.8499 | 729.5464 | 705.0881 | 0.648648 |  |
| GLIPR2 | 212.3058 | 243.9193 | 219.982 | 144.6598 | 128.2842 | 128.0101 | 0.754179 |  |
| HDAC7 | 3697.049 | 3420.99 | 3460.486 | 4658.839 | 5619.962 | 4933.552 | -0.52408 |  |
| NRP2 | 161.0596 | 212.4459 | 119.3919 | 65.39416 | 75.85498 | 55.74635 | 1.326119 |  |
| MYO1C | 13943.16 | 14069.51 | 13628.54 | 19383.42 | 20961.63 | 20552.85 | -0.54836 |  |
| BMP4 | 12860.71 | 14866.84 | 10090.03 | 21110.43 | 23276.32 | 22171.56 | -0.81553 |  |
| FGF9 | 32.42108 | 61.1984 | 59.22592 | 139.7057 | 168.4427 | 160.0127 | -1.60597 |  |
| SEMA4C | 2116.783 | 1879.665 | 1899.93 | 3394.552 | 3193.718 | 3076.373 | -0.71323 |  |
| ANXA1 | 10571.36 | 11702.01 | 10654.08 | 7468.806 | 8615.118 | 7872.623 | 0.458975 |  |
| NRP1 | 441.345 | 314.7346 | 429.6229 | 611.3364 | 564.4503 | 678.2472 | -0.64671 |  |
| ITGA3 | 9523.431 | 10542.74 | 9458.285 | 10914.88 | 13742.02 | 13217.05 | -0.35922 |  |
| PTP4A1 | 23.00851 | 17.48526 | 17.86178 | 3.963283 | 6.693087 | 4.129359 | 1.98724 |  |
| ITGA2B | 16.73346 | 17.48526 | 20.68207 | 47.55939 | 46.85161 | 35.09955 | -1.2367 |  |
| PDGFC | 80.52978 | 119.774 | 92.1292 | 42.60529 | 53.54469 | 58.84337 | 0.920529 |  |
| SOX9 | 11798.14 | 11150.35 | 13075.77 | 14453.1 | 16121.41 | 16047.72 | -0.37204 |  |
| SEMA6C | 323.165 | 283.2612 | 313.9914 | 418.1263 | 489.7108 | 405.7095 | -0.5133 |  |
| SEMA6B | 1613.733 | 1827.209 | 1399.8 | 2042.081 | 2524.409 | 2142.105 | -0.47039 |  |
| HYAL1 | 121.3176 | 109.2829 | 103.4103 | 53.50432 | 62.46881 | 71.23144 | 0.83513 |  |
| BMPR2 | 1822.901 | 1703.064 | 1677.128 | 2388.869 | 2089.359 | 2321.732 | -0.38654 |  |
| PLK2 | 246.8185 | 396.0411 | 236.9037 | 484.5113 | 493.0574 | 437.7121 | -0.68363 |  |
| SCARB1 | 3589.327 | 4310.99 | 4017.021 | 4781.701 | 5420.285 | 5875.046 | -0.43173 |  |
| FGF18 | 219.6267 | 149.4989 | 204.9405 | 310.1269 | 310.113 | 323.1223 | -0.71956 |  |
| SEMA3B | 10257.61 | 5443.16 | 10000.72 | 14156.85 | 13117.33 | 15812.35 | -0.74548 |  |
| HCT116 | | | | | | | | |
| gene_name | NTC_U_1 | NTC_U_2 | NTC_U_3 | IKO_U_1 | IKO_U_2 | IKO_U_3 | log2FoldChange | |
| **PTAFR** | **135.5125** | **142.7644** | **104.0422** | **350.7722** | **179.3428** | **256.7571** | **-1.043739605** | |
| **NR4A3** | **28.52894** | **31.45657** | **38.8729** | **87.18616** | **83.57721** | **160.1423** | **-1.733884397** | |
| **GATA2** | **2515.896** | **1967.245** | **1884.192** | **5966.507** | **5265.364** | **5476.603** | **-1.391203979** | |
| **ETS1** | **501.931** | **543.2307** | **495.0578** | **281.1585** | **115.7893** | **179.9947** | **1.410130713** | |
| IGFBP5 | 52.60023 | 31.45657 | 37.72958 | 91.91719 | 55.71814 | 67.498 | -0.81655 | |
| CSF1 | 59.73247 | 71.38221 | 59.45266 | 158.8275 | 112.3069 | 115.1437 | -1.02924 | |
| GPER1 | 202.3772 | 186.3197 | 157.7782 | 314.9516 | 361.2973 | 383.8122 | -0.95017 | |
| AKT3 | 304.0115 | 333.9236 | 300.6933 | 685.9996 | 464.8982 | 622.0404 | -0.91851 | |
| SEMA3A | 10.69835 | 9.678944 | 8.003243 | 121.6551 | 102.7303 | 99.26177 | -3.50544 | |
| LGR6 | 37.44423 | 16.93815 | 42.30286 | 6.758617 | 11.31775 | 13.2349 | 1.666409 | |
| F2RL1 | 4906.086 | 5631.936 | 5126.649 | 11786.35 | 8385.58 | 11322.46 | -1.00766 | |
| STK39 | 6862.993 | 7251.949 | 6799.327 | 6289.569 | 2609.176 | 4082.967 | 0.687655 | |
| GLIPR2 | 1220.504 | 1355.052 | 940.9527 | 847.5306 | 981.1616 | 962.1774 | 0.334289 | |
| HDAC7 | 2996.43 | 2707.685 | 3256.177 | 2433.102 | 2186.937 | 2218.17 | 0.389601 | |
| NRP2 | 68.64776 | 55.65393 | 52.59274 | 180.4551 | 114.9187 | 99.26177 | -1.16328 | |
| MYO1C | 10134.01 | 10115.71 | 9388.948 | 11320.68 | 12308.48 | 12041.11 | -0.26716 | |
| BMP4 | 10097.46 | 9191.367 | 6890.792 | 11470.72 | 14205.51 | 11172.9 | -0.49314 | |
| FGF9 | 91.82752 | 61.70327 | 35.44293 | 38.52412 | 33.95324 | 27.7933 | 0.910342 | |
| SEMA4C | 2857.352 | 2750.03 | 2699.38 | 2365.516 | 1811.71 | 1817.152 | 0.469586 | |
| ANXA1 | 5043.382 | 4827.373 | 5160.949 | 5665.073 | 6290.055 | 6408.34 | -0.28853 | |
| NRP1 | 156.0176 | 140.3447 | 171.4981 | 819.8202 | 319.5087 | 782.1827 | -2.03712 | |
| ITGA3 | 12014.25 | 10951.73 | 12375.3 | 25465.79 | 14055.77 | 21605.98 | -0.79047 | |
| PTP4A1 | 47.25106 | 39.92564 | 40.01622 | 283.8619 | 163.672 | 230.2873 | -2.41024 | |
| ITGA2B | 537.5922 | 476.688 | 507.6343 | 475.8066 | 296.0026 | 308.3732 | 0.489542 | |
| PDGFC | 271.0249 | 257.7019 | 258.3904 | 436.6067 | 327.3441 | 422.1934 | -0.59048 | |
| SOX9 | 6007.125 | 6334.869 | 5540.531 | 3070.44 | 3702.644 | 3576.071 | 0.789435 | |
| SEMA6C | 1398.81 | 1192.93 | 1442.87 | 996.896 | 819.2307 | 874.8271 | 0.583614 | |
| SEMA6B | 5291.227 | 4535.795 | 4545.842 | 6266.59 | 5845.181 | 6228.345 | -0.35138 | |
| HYAL1 | 225.5569 | 216.5664 | 221.8042 | 677.8893 | 336.9206 | 697.4794 | -1.36524 | |
| BMPR2 | 1644.872 | 1928.53 | 1830.456 | 3436.081 | 2955.673 | 3373.577 | -0.85415 | |
| PLK2 | 2237.739 | 2059.195 | 1629.232 | 3304.964 | 3687.844 | 2691.979 | -0.70885 | |
| SCARB1 | 3832.685 | 4610.807 | 3744.375 | 5875.266 | 5202.681 | 6581.717 | -0.5349 | |
| FGF18 | 45.468 | 52.02432 | 30.86965 | 131.1172 | 70.51827 | 96.61479 | -1.22088 | |
| SEMA3B | 1618.126 | 1480.878 | 1681.824 | 6688.327 | 3957.729 | 5238.374 | -1.73239 | |

Table S5：Association between GATA2 expression and clinicopathological features of patients with colorectal cancer

|  |  | IRF5 expression | |  | |  |
| --- | --- | --- | --- | --- | --- | --- |
|  | Total | Low | High |  | |  |
|  | (n = 40) | (n = 20) | (n = 20) | *P* value | |  |
| Gender |  |  |  | 0.7515 | |  |
| Male | 21 | 11 | 10 |  | |  |
| Female | 19 | 9 | 10 |  | |  |
| Age(year) |  |  |  | 0.5250 | |  |
| <60 | 18 | 10 | 8 |  | |  |
| ≥60 | 22 | 10 | 12 |  | |  |
| T stage |  |  |  | 0.0110* | |  |
| T1-T2 | 18 | 13 | 5 |  | |  |
| T3-T4 | 22 | 7 | 15 |  | |  |
| N stage |  |  |  | 0.0064** | |  |
| N0 | 17 | 13 | 4 |  | |  |
| N1+N2+N3 | 22 | 6 | 16 |  | |  |
| M stage |  |  |  | 0.0090** | |  |
| M0 | 25 | 17 | 8 |  | |  |
| M1 | 15 | 3 | 12 |  | |  |
|  |  |  |  |  |  |  |
| ^*^*P*<0.05，^*^*P*<0.01 | | |  |  |  | |
